# A Translational Pharmacokinetic Rat Model of Cerebral Spinal Fluid and Plasma Concentrations of Cefepime

**DOI:** 10.1128/mSphere.00595-18

**Published:** 2019-01-30

**Authors:** Sean N. Avedissian, Gwendolyn Pais, Medha D. Joshi, Nathaniel J. Rhodes, Marc H. Scheetz

**Affiliations:** a Department of Pharmacy Practice, Chicago College of Pharmacy, Midwestern University, Downers Grove, Illinois, USA; b Center of Pharmacometric Excellence, Midwestern University, Downers Grove, Illinois, USA; c Department of Pharmaceutical Sciences, Chicago College of Pharmacy, Midwestern University, Downers Grove, Illinois, USA; d College of Graduate Studies, Department of Pharmacology, Midwestern University, Downers Grove, Illinois, USA; Antimicrobial Development Specialists, LLC

**Keywords:** cefepime, cerebral spinal fluid, pharmacokinetics

## Abstract

This study sought to define the transit of cefepime between plasma and cerebral spinal fluid (CSF) in a rat model. Male Sprague-Dawley rats received cefepime intravenously. A total daily dose of 150 mg/kg of body weight/day was administered as a single injection every 24 h for 4 days. Plasma samples were obtained via a second dedicated intravenous catheter. CSF sampling occurred via an intracisternal catheter. Cefepime levels in plasma and CSF were quantified via liquid chromatography-tandem mass spectrometry (LC-MS/MS). Pharmacokinetic (PK) analyses were conducted using Pmetrics for R. PK parameters and exposures during the first 24 h (i.e., area under the concentration-time curve from 0 to 24 h [AUC_0–24_] and maximum concentration of drug in serum from 0 to 24 h [*C*_max 0–24_]) were calculated from Bayesian posteriors. CSF penetration was estimated by comparing the exposure profiles between plasma and the CSF. Eleven rats contributed PK data. A four-compartmental model with a lag compartment for CSF fit the data well for both plasma (Bayesian [*R*^2^ = 0.956]) and CSF (Bayesian [*R*^2^ = 0.565]). Median parameter values (with the coefficient of variation percentage [CV%] in parentheses) for the rate constants to CSF from the lag compartment (*K*_34_), to the central compartment from the CSF compartment (*K*_41_), and to the lag compartment from the central compartment (*K*_13_) were 2.96 h^−1^ (116.27%), 0.47 h^−1^ (54.86%), and 0.13 h^−1^ (23.42%), respectively. The elimination rate constant (*k*_el_) was 3.15 h^−1^ (7.5%). Exposure estimation revealed a plasma median (with interquartile range [IQR] in parentheses) half-life, AUC_0–24_, and *C*_max 0–24_, of 1.7 (1.5 to 1.9) h, 111.3 (95.7 to 136.5) mg · 24 h/liter, and 177.8 (169.7 to 236.4) μg/ml, from the first dose, respectively. Exposure estimation of CSF demonstrated a median (with IQR in parentheses) AUC_0–24_ and *C*_max 0–24_ of 26.3 (16.6 to 43.1) mg · 24 h/liter and 6.8 (5.2 to 9.4) μg/ml, respectively. The median CSF/blood percentage of penetration was 19%. Cefepime transit to the CSF is rapid and predictable in the rat model. This model will be highly useful for understanding the therapeutic window for cefepime and neurotoxicity.

**IMPORTANCE** This study defines the transit of cefepime between plasma and cerebral spinal fluid (CSF) in a rat model. Male Sprague-Dawley rats received cefepime intravenously. Plasma samples were obtained via a second dedicated intravenous catheter. CSF sampling occurred via an intracisternal catheter. Drug exposures and transfer from the plasma to the CSF during the first 24 h were calculated. The median CSF/blood percentage of penetration was 19%. Cefepime transit to the CSF is rapid and predictable in the rat model. This model will be highly useful for understanding the therapeutic window for cefepime and neurotoxicity.

## Abstract (precis)

This study defines the transit of cefepime between plasma and cerebral spinal fluid (CSF) in a rat model. Male Sprague-Dawley rats received cefepime intravenously. Plasma samples were obtained via a second dedicated intravenous catheter. CSF sampling occurred via an intracisternal catheter. Drug exposures and transfer from the plasma to the CSF during the first 24 h were calculated. The median CSF/blood percentage of penetration was 19%. Cefepime transit to the CSF is rapid and predictable in the rat model. This model will be highly useful for understanding the therapeutic window for cefepime and neurotoxicity.

## INTRODUCTION

Antibiotic-resistant Gram-negative bacillus (GNB) infections are characterized by high morbidity and mortality. These infections have reached epidemic proportions—infecting at least 2 million Americans annually and resulting in 23,000 attributable deaths (1). These infections represent an “urgent and serious” threat (1) and have led to a Presidential Executive Order and National Action Plan to combat the threat (2, 3). One methodology to mitigate poor GNB outcomes is to use maximal antimicrobial exposures; however, this leaves toxicity unconsidered. β-Lactams are highly effective against GNB; optimal exposure is mediated when concentrations remain above the MIC of GNB for prolonged periods of time. Elevated MICs for GNB now mean that standard FDA-approved doses of β-lactams may no longer be effective. Our previous studies have examined exposure thresholds and defined several clinical failure thresholds for β-lactam agents according to increasing MIC (4–6).

Similar to other β-lactams, the pharmacokinetic/pharmacodynamic (PK/PD) index most predictive of clinical outcomes for cefepime is the time fraction of unbound drug above the MIC of the pathogen (∫*T*_>MIC_ [∼60 to 70% ∫*T*_>MIC_]) (6, 7). Current guideline recommendations for management of bacterial meningitis list cefepime as a guideline-approved therapy (8). Faced with rising MICs, many clinicians use maximal cefepime exposures to cure these infections and prevent resistance. However, using higher doses in an unguided manner has led to excess neurotoxicity (9–11), as also documented by an FDA safety announcement in 2012 (12). Despite the knowledge that higher doses seem to cause neurotoxicity, the exact exposure index mediating cefepime-associated neurotoxicity remains unclear (5).

Several animal and human studies have evaluated and described the transit of cefepime from the central distribution (i.e., plasma) to the target areas (i.e., brain and cerebral spinal fluid [CSF]) (13–15). Human studies put cefepime penetration between a median of 8% and mean of 23% (13), and animal models have demonstrated cefepime CSF concentrations between 16.2 and 36% (14, 15). However, there is very little information about the real-time transfer of cefepime from the blood to the CSF from studies with robust samples. Quantitatively defining this relationship will ultimately be required to fully understand the exposure drivers of neurotoxicity. Thus, the objective of the proposed research was 2-fold: (i) to explain cefepime transit from the plasma to the CSF and (ii) to estimate the percentage of cefepime crossing from plasma to CSF in a rat model.

## RESULTS

### Characteristics of animal cohort.

A total of 11 rats received cefepime and had plasma and CSF concentrations sampled. All rats had nine plasma concentrations (complete sampling) and a median of six CSF concentrations sampled over the 4-day protocol. Four animals had intracisternal catheter failures before the collection of a single CSF sample. All plasma samples collected were utilized for model building, with the exception that one rat had a single plasma concentration 100-fold higher than anticipated (i.e., 5,082 μg/ml); this concentration was excluded from analysis.

### Cefepime PK models.

**FIG 1:**
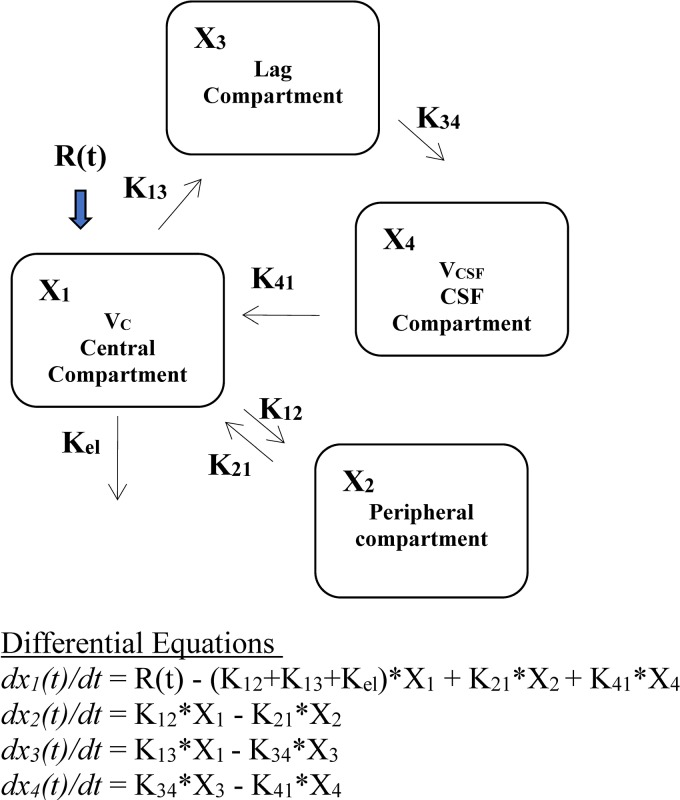
Schematic and differential equations of base four-compartmental PK model with a lag compartment for CSF. Abbreviations: PK, pharmacokinetic; K_el_, elimination rate constant; V_c_, volume of central compartment; V_CSF_, volume of CSF compartment; K_12_, rate constant to peripheral from central compartment; K_21_, rate constant to central from peripheral compartment; K_13_, rate constant to lag compartment from central compartment; K_34_, rate constant to CSF from lag compartment; K_41_, rate constant to central from CSF compartment; X_1_, amount in the central compartment; X_2_, amount in the peripheral compartment; X_3_, amount in the lag compartment; X_4_, amount in the CSF compartment.

**FIG 2:**
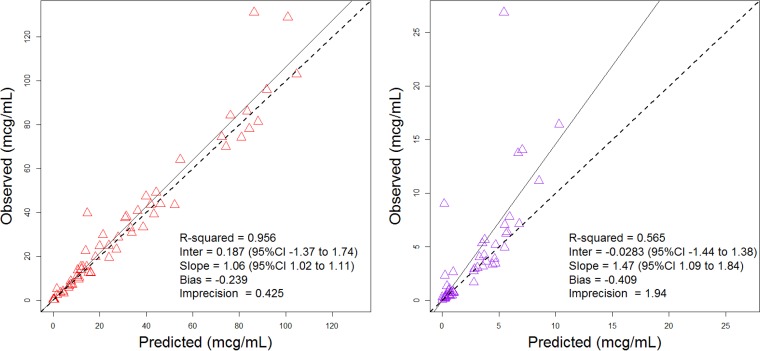
Observed versus predicted Bayesian plots from the final model for plasma (left) and CSF (right). CI, confidence interval.

Models successfully converged for all two-, three-, and four-compartment models. The final model was a four-compartment model with a lag compartment for CSF (Fig. 1) with an Akaike information criterion (AIC) score of 509 (Table 1). The final model’s median parameter values for elimination rate constant (*k*_el_), volume of central compartment (*V_c_*), volume of CSF compartment (*V*_CSF_), the rate constant to the peripheral from the central compartment (*K*_12_), the rate constant to the central from the peripheral compartment (*K*_21_), the rate constant to the CSF compartment from the lag compartment (*K*_34_), the rate constant to the central compartment from the CSF compartment (*K*_41_), and the rate constant to the lag compartment from the central compartment (*K*_13_) were as follows (with the coefficient of variation percentage [CV%] in parentheses): *k*_el_, 3.15 h^−1^ (7.52%); *V_c_*, 0.11 liter (22.9%); *V*_CSF_, 0.14 liter (64.4%); *K*_12_, 18.20 h^−1^ (40.17%); *K*_21_, 41.98 h^−1^ (10%); *K*_34_, 2.96 h^−1^ (116.27%); *K*_41_, 0.47 h^−1^ (54.86%); and *K*_13_, 0.13 h^−1^ (23.42%) (Table 2). In the model for predictive performance of observed versus Bayesian predicted concentrations, bias, imprecision, and the coefficient of determination (*R*^2^) were, respectively, −0.239 μg/ml, 0.425 (μg/ml)^2^, and 0.956 for plasma and −0.409 μg/ml, 1.94 (μg/ml)^2^, and 0.565 for CSF (Fig. 2).

**TABLE 1:** PK model build comparison^*a*^

Model	−2LL	AIC	Result for Bayesian parameter in compartment					
			Central			CSF		
			Bias (μg/ml)	Imp (μg/ml)	*R*^2^	Bias (μg/ml)	Imp (μg/ml)	*R*^2^
2-compartment	526	538.6	−0.22	0.44	0.93	−0.13	3.87	0.34
3-compartment	521.9	539	−0.29	0.38	0.93	−0.63	1.64	0.47
3-compartment with lag constant (*K*_13_)	523.4	538.2	−0.19	0.42	0.93	−0.95	1.84	0.48
4-compartment with lag compartment (*K*_13_ and *K*_34_)^*b*^	509	528.3	−0.23	0.43	0.96	−0.41	1.94	0.57

a Abbreviations: PK, pharmacokinetic; *K*_13_, rate constant to lag compartment from central compartment; *K*_34_, rate constant to CSF compartment from lag compartment; −2LL, −2 log likelihood; AIC, Akaike information criterion; Imp, imprecision; CSF, cerebral spinal fluid.

b Final model based on regression of observed versus predicted concentrations, visual plots of parameter estimates, lowest AIC, and rule of parsimony.

**TABLE 2:** Median parameter values from final model^*a*^

PK parameter	Median	CV%	Variance
*k*_el_ (h^−1^)	3.15	7.5	0.059
*V_c_* (liters)	0.11	22.9	0.001
*V*_CSF_ (liters)	0.14	64.4	0.014
*K*_12_ (h^−1^)	18.20	40.2	45.55
*K*_21_ (h^−1^)	41.98	10	16.668
*K*_13_ (h^−1^)	0.13	23.4	0.001
*K*_34_ (h^−1^)	2.96	116.3	180.807
*K*_41_ (h^−1^)	0.47	54.9	0.101

a Abbreviations: PK, pharmacokinetic; CV%, coefficient of variation percentage; *k*_el_, elimination rate constant; *V_c_*, volume of central compartment; *V*_CSF_, volume of cerebral spinal fluid compartment; *K*_12_, rate constant to peripheral compartment from central compartment; *K*_21_, rate constant to central compartment from peripheral compartment; *K*_13_, rate constant to lag compartment from central compartment; *K*_34_, rate constant to cerebral spinal fluid compartment from lag compartment; *K*_41_, rate constant to central compartment from cerebral spinal fluid compartment.

### Cefepime PK exposures and percentage of CSF penetration.

The overall pharmacokinetic exposures for all rats are summarized in Table 3. PK median parameters for plasma were as follows (with the interquartile range [IQR] in parentheses): half-life of 1.7 (1.5 to 1.9) h, AUC_0–24_ of 111.3 (95.7 to 136.5) mg · h/liter, and *C*_max 0–24_ of 177.8 (169.7 to 236.4) μg/ml, from the first dose. PK exposures revealed a CSF median (IQR) AUC_0–24_ of 26.3 (16.6 to 43.1) mg · h/liter and *C*_max 0–24_ of 6.8 (5.2 to 9.4) μg/ml. The median percentages of cefepime penetration into the CSF were 19% as calculated from AUC_0–24_ and 3% as calculated from *C*_max 0–24_. The complete list of percentages of cefepime penetration into CSF for each animal can be found in Table 3.

**TABLE 3:** Cefepime plasma and CSF PK exposures estimated using Bayesian posteriors for AUC_0–24_ and *C*_max 0–24_ and percentage of cefepime in CSF or blood^*a*^

Animal	*C*_max 0–24_ (μg/ml) for plasma^*b*^	AUC_0–24_ (mg · h/liter) for plasma	*C*_max 0–24_ (μg/ml) for CSF	*T*_max_ (h) for CSF	AUC_0–24_ (mg · h/liter) for CSF	*t*_1/2_ (h)	% penetration for CSF/plasma by:	
							*C*_max 0–24_	AUC_0–24_
1	169.7	92.8	3.8	1.2	16.6	2.2	2	18
2	433.4	163.5	8.5	0.4	10.5	0.6	2	6
3^*c*^	170	108.6	12	2	51.0	1.4	7	47
4	197.4	112.4	3.6	1	13.7	1.9	2	12
5^*c*^	251.7	145.8	6.4	1.8	28.0	1.9	3	19
6^*c*^	205.6	123.0	6.8	1.8	29.8	1.8	3	24
7	236.4	136.5	6	1.8	26.3	1.9	3	19
8	153.4	95.7	9.4	2	43.1	1.7	6	45
9	177.8	100.1	7.1	0.9	21.9	1.5	4	22
10^*c*^	167.5	94.8	5.2	1.2	18.5	1.7	3	20
11	173.1	111.3	11.7	2	51.2	1.5	7	46
Median (IQR)								
All animals	177.8 (169.7–236.4)	111.3 (95.7–136.5)	6.8 (5.2–9.4)	1.8 (1–2)	26.3 (16.6–43.1)	1.7 (1.5–1.9)	3 (2–6)	20 (18–45)
Excluding animals^*c*^	177.8 (169.7–236.4)	111.3 (95.7–136.5)	7.1 (3.8–9.4)	1.2 (0.9–2)	21.9 (13.7–43.1)	1.7 (1.5–1.9)	3 (2–6)	19 (12–45)

a Abbreviations: *C*_max 0–24_, maximum concentration at 24 h; AUC_0–24_, area under the curve at 24 h; CSF, cerebral spinal fluid; *T*_max_, time to maximal concentration; *t*_1/2_, half-life; IQR, interquartile range.

b The *C*_max_ in the central compartment was estimated from the Bayesian posterior profiles for each animal in 12-min intervals (i.e., *T*_max_ = 0.2 h for all animals).

c No CSF samples were obtained in this animal, and CSF PK exposures were estimated using the final model.

## DISCUSSION

To our knowledge, this is the first study that quantitatively describes the transit of cefepime from plasma to the CSF at the individual animal level and over a period of several days. These data are necessary to develop physiologically based pharmacokinetic models and further enhance translation. Obtaining individual-level pharmacokinetics required multiple samplings of both blood and CSF over the course of study. Previous studies analyzed population-level (i.e., single samples of blood and CSF pooled over a population of animals) PK in neonatal rats (15) or meningitis rabbit models (14, 16). Gerber et al. and Tauber et al. obtained serial concentrations of plasma and CSF from rabbits over 7 to 8 h, but continual anesthesia was required for the entire experimental period (14, 16). Collectively, these studies found that cefepime CSF penetration varied from 16.2% to 36%, depending on the animal model utilized (rats versus rabbits) and if inflammation/infection was present (14, 15). We found that the median percentage of penetration of cefepime calculated by the ratio of AUC_0–24_ in CSF to plasma was 19%, and collectively the results from animal studies agree with results from human studies (range of 8 to 23%) (13–15, 17). The agreement between the human and animal models provides high translational capacity, and our model further limits the number of animals required in the study (as noninvasive repeat samples are readily possible, and population averaging per time point is not required). These findings are highly important to the movement to reduce and refine humane animal research (18).

Rodent models have been utilized for neurodegenerative diseases for many years given they possess similar brain structure connectivity and release similar neurotransmitters. Furthermore, the rat has been used in many phenotypic seizure models (19). Additionally, enhancement of cellular glutamate uptake via glutamate transporter subtype 1 (GLT-1) in the rat, which is named EAAT2 (excitatory amino acid transporter-2) in humans, has been studied for other β-lactams (i.e., ceftriaxone) (20). Less is known about cefepime. Further study is warranted.

For translational purposes, it is important to note that our study utilized total drug concentrations of cefepime. As free concentrations were not measured, we did not employ a population value to correct for protein binding as this could be potentially misleading. Notably, human plasma protein binding for cefepime is low (∼10 to 20% [21–24]), and binding in rats is lower than that in humans for other β-lactams (24); thus, most drug is expected to be free in both models. Additionally, since CSF volume in the rat is very low, it is technically difficult to quantify the free concentration in the CSF (which theoretically would be higher than the plasma). These results would be expected to increase penetrance values; however, confirmatory work would be needed to classify free drug penetrance.

We identified that peak concentrations in the CSF are blunted and produce different estimates of penetration compared to AUC-based evaluations. The latter is more relevant in our opinion as β-lactams are time-dependent drugs pharmacodynamically, and this has been the standard for defining cefepime penetration in the literature (25). Our analysis also demonstrates that the mass transit of cefepime to the CSF occurs rapidly (i.e., *K*_13_ + *K*_34_ = 3.09 h^−1^), indicating that steady state is approached within an hour (0.693/3.09 × 3 = 0.67 h). This finding is similar to results from a previous study by Lodise and colleagues that described the mass transit of cefepime in 7 patients with external ventricular drains (13). Their estimation of the intercompartmental rate constant (*K*_13_) in their study was nearly identical (i.e., 3.4 h^−1^). Thus, the rat model highly replicates the human condition. It should be noted that the predicted median *t*_1/2_ of 1.7 h was calculated from the terminal elimination phase as described in Materials and Methods. When *t*_1/2_ is calculated from *k*_el_ in our model, a substantially different result is obtained [i.e., ln(2)/median *k*_el_ = 0.22 h]. The compartmental based *t*_1/2_ calculated is more relevant to acute elimination phase, and those wishing to utilize calculations from our study should understand the differences in calculations for their own application.

**FIG 3:**
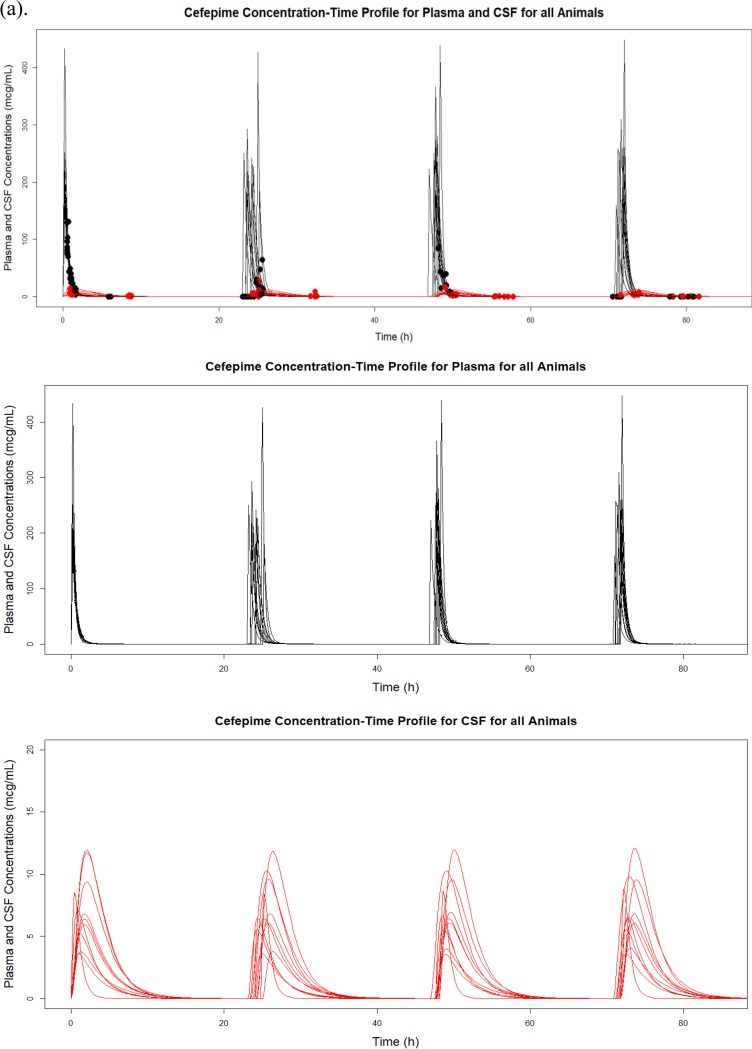

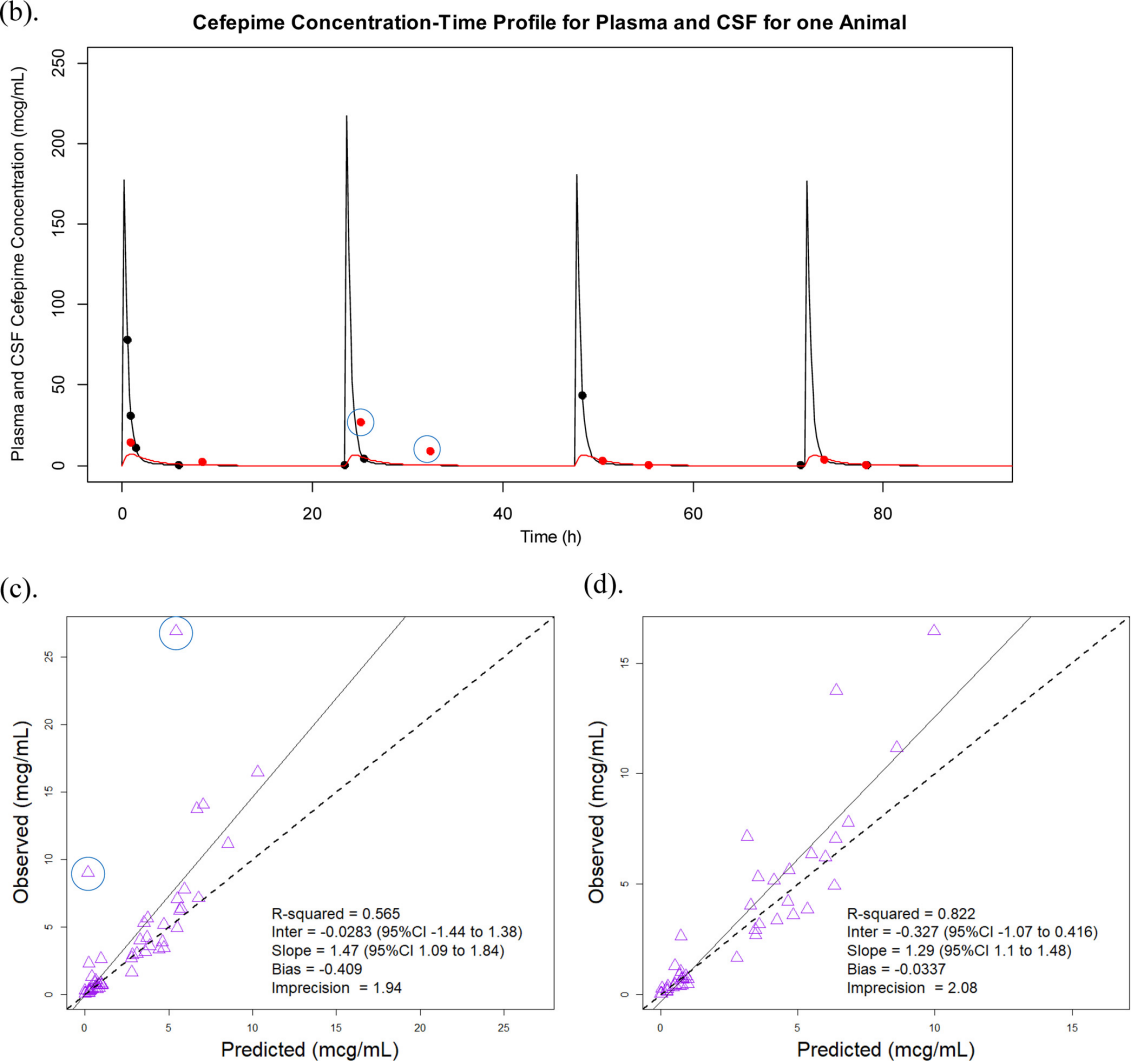
Plasma (black) and CSF (red) Bayesian observed versus predicted plots for all animals (a) and one animal (b) with two observed CSF concentrations higher than predicted (circled) and overall improvement in CSF observed versus predicted Bayesian plots before (c) and after (d) an animal is excluded.

We identified two potential CSF concentration outliers in one animal during the evaluation of each animal’s pharmacokinetic profile (Fig. 3a and b). This particular profile suggested that the animal may have experienced higher than normal exposure to cefepime in CSF due to potential inflammation or a blood-contaminated tap, although we conservatively opted to keep this animal in the overall analysis. Model fits were substantially better without the inclusion of this animal (Fig. 3c and d); however, as we were unable to verify the cause of the discrepancy, these values were used in our final model.

We acknowledge several limitations in our study exist. First, this study protocol utilized a single daily dose of cefepime. It is unknown if concentration-mediated changes to CSF transit occur. Unfortunately, assessment of dose response was beyond the scope of this pilot study. It remains possible that transit rate and extent are driven by concentration: thus, magnitude of dose may influence central nervous system (CNS) penetration. This would require further study to explore; however, this has not been the case for the majority of β-lactams studied clinically. Second, animals displayed a CV% of <50% (values not shown) across exposure measurements (i.e., AUC and *C*_max_), demonstrating moderate interanimal variability. These results substantiate the need to study PK at the individual animal level and that variability is similar to that of human studies. Third, four animals had occluded intracisternal catheters before the experimental portion of the study began, and CSF could not be sampled; however, seven animals had more complete data, with ≥4 CSF samples each. Fourth, our model did not utilize infected animals where inflamed meninges can increase the amount of cefepime that reaches the CSF (14, 15). However, the results of our model estimates for mass transit from plasma to CSF are numerically similar to the estimation by Lodise et al. (13). Finally, one animal displayed a plasma concentration of 5,082 μg/ml, which was over 100-fold higher than expected. This concentration was omitted. A potential explanation for this occurrence was that the sample might have been taken from a syringe that was used to administer the cefepime dose given the proximity of the sample to dose administration to the animal.

In summary, these data demonstrate that cefepime transit to the CSF is rapid and highly predictable in the rat model. The outcomes from the rat model agree highly with those from human studies. This rat model will be highly useful for future applications, including understanding neurotoxicity outcomes for cefepime. Further studies are warranted.

## MATERIALS AND METHODS

This pharmacokinetic/toxicodynamic (PK/TD) study was conducted at Midwestern University in Downers Grove, IL. All study methods were approved by the Institutional Animal Care and Use Committee (IACUC; protocol no. 2793) and conducted in an AAALAC-accredited animal facility.

### Experimental design and animals.

Male Sprague-Dawley rats (*n* = 11, mean weight, 306 g) were obtained from Charles River (Raleigh, NC). All catheters (cisternal and vein cannulation) for the animals were surgically implanted (26, 27) at Charles River prior to shipping. On arrival at the housing facility, animals were acclimated prior to starting the study protocol. Animals were administered 150 mg/kg cefepime daily for 4 days as a single daily dose (as described below). All cefepime doses were administered through intravenous (i.v.) injection over 2 min via a dedicated internal jugular vein catheter. The dose chosen for this study allometrically scales to a humanized infusion of 24 mg/kg (28). Rats were housed in a light- and temperature-controlled room for the duration of the study and allowed free access to water and food, except during sampling. Data were analyzed for all animals that entered a protocol. When animals contributed incomplete data (i.e., early protocol termination), all available cefepime levels were analyzed for PK model build.

### Chemicals and reagents.

Animals were administered cefepime hydrochloride (Hospira, Lake Forest, IL [lot no. 144G005]) for injection. Artificial CSF (Tocris Bioscience, Bristol, UK) and normal saline (Abbott [lot no. C950212]) were used as in the sampling methods described below. Liquid chromatography-tandem mass spectrometry (LC-MS/MS) standard curves were generated using commercially obtained cefepime hydrochloride (USP) (Chem-Impex International, Inc., Wood Dale, IL) with a purity of greater than 99.5%. Ceftazidime pentahydrate (Acros Organics, NJ) was used as an internal standard for the cefepime quantification. Formic acid was obtained from Thermo Fisher Scientific (Waltham, MA). Acetonitrile (BDH [lot no. 16I011254]) and methanol (BDH [lot no. 16L204024]) were obtained from VWR Analytical (Chicago, IL). Milli-Q water was obtained from Midwestern University via an Aqua Solutions water purification dispensatory. Frozen, nonmedicated, nonimmunized, pooled Sprague-Dawley rat plasma (anticoagulated with disodium EDTA) and CSF were used for calibration of standard curves (BioreclamationIVT, Westbury, NY).

### Blood and CSF sampling.

Blood samples were drawn from a single right-sided internal jugular vein catheter in a sedation-free manner when possible. CSF was collected via an intracisternal catheter. Isoflurane gas was used for temporary sedation when needed (5% initially, followed by 1 to 3% maintenance). Within the first 24 h, the target samplings were *n* = 5 blood and *n* = 2 CSF samples per animal. The full sampling strategy over the 4-day study can be found in Table 4.

**TABLE 4:**
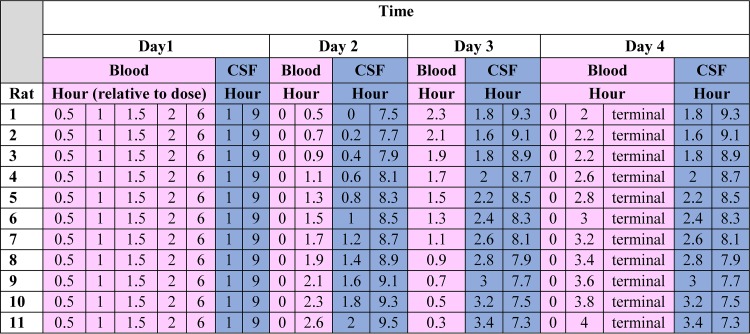
Example of the staggered sampling schematic utilized for the study protocol^*a*^

a CSF, cerebral spinal fluid.

Each sample (0.25 ml blood and 0.05- to 0.1-ml CSF aliquots) was replaced with an equivalent volume of either normal saline or artificial CSF (as appropriate) to maintain euvolemia. Blood and CSF samples from cefepime-treated animals were processed similarly to previous reports (29, 30).

### Determination of cefepime concentrations in plasma and CSF.

Plasma and CSF concentrations of cefepime were quantified by LC-MS/MS using individual standard curves for each matrix. Milli-Q water containing 0.1% formic acid and acetonitrile (flow rate of 0.5 ml/min) were used as aqueous (A) and organic (B) solvents, respectively, at the following ramping transitions: 0.00 min A (90%) → B (10%), 1.50 min A (90%) → B (10%), 2.50 min A (10%) → B (90%), 5.40 min, A (10%) → B (90%), 5.50 min A (90%) → B (10%), and 10 min A (90%) → B (10%). Plasma and CSF sample volumes of 40 μl were combined with 4 μl of internal standard (10 μg/ml ceftazidime) and subjected to protein precipitation using 455 μl methanol plus 1% formic acid. Following centrifugation for 10 min at 16,000 × *g* (Eppendorf model 5424; Eppendorf AG, Barkhausenweg, Germany), 75 μl of the supernatant was collected and reserved for analysis. A Poroshell (3.0 × 100 mm, 2.7 μm) 120 EC-C_18_ column (Agilent Technologies, Inc., Santa Clara, CA) was utilized, with the column temperature held at 25°C for both analyses. Two microliters was injected into an Agilent 1260 LC attached to Agilent 6420 triple quadrupole mass spectrometer. Mass spectrometry analysis was conducted with electrospray ionization in positive mode (ESI^+^). The MS source conditions were as follows: gas temperature set to 350°C, gas flow set to 13 liters/min, nebulizer set at 40 lb/in^2^, fragmentor set at 68 and 78 V (for ceftazidime and cefepime, respectively), and cell accelerator voltage set at 4 V. Collision energies for the cefepime quantifier and qualifier were set at 30 and 10 eV. Ceftazidime collision energy was set at 10 eV. The following transitions (*m*/*z*) for cefepime and ceftazidime were identified and utilized: 241.1 → 84.1 for cefepime quantifier, 241.1→86.1 for cefepime qualifier, and 274.1 → 80.2 for cefepime quantifier. A weight of 1/x was applied to the regression analysis (31, 32). The assay was linear between plasma concentrations of 0.5 and 60 μg/ml (*R*^2^ = 0.998) and CSF concentrations of 0.125 and 40 μg/ml (*R*^2^ = 0.99). Precision was <7.6% for all measurements, including intra- and interassay measurements. Greater than 92% of the analyte was recovered in all samples tested, with overall mean assay accuracies of 100% for plasma and 98% for CSF. Any samples measuring above the upper limit were diluted with corresponding matrix so that the analyzed concentration was within range of the standard curve.

### Cefepime PK model.

The simplest base model considered was a 2-compartment model with a plasma compartment and a CSF compartment. Three- and four-compartment models with/without a lag constant were similarly fit (as described below) using the nonparametric adaptive grid (NPAG) algorithm within the Pmetrics package version 1.5.0 (Los Angeles, CA) for R version 3.2.1 (R Foundation for Statistical Computing, Vienna, Austria) (33, 34). The initial estimate of parameter weighting was accomplished using the inverse of the assay variance. The observation variance was proportional with a multiplicative (γ) model (error = standard deviation [SD] × γ) where SD = *C*_0_ + *C*_1_*Y* (with inputs for plasma at *C*_0_ = 0.25 and *C*_1_ = 0.15 and CSF at *C*_0_ = 0.0625 and *C*_1_ = 0.15 ng/ml and where *Y* = observed concentration). Model performance was quantitatively described using observed versus predicted concentrations to calculate bias, imprecision, and coefficients of determination (35). The final model was selected according to the lowest Akaike information criterion (AIC) score and the rule of parsimony.

### Estimation of PK exposure and percentage of CSF penetration.

The best-fit model was utilized to obtain median maximum *a posteriori* probability (MAP) Bayesian cefepime plasma and CSF concentration estimates at 12-min intervals over the 24-h study period using each animal’s measured cefepime concentrations, exact dose, and dosing schedule. Bayesian posteriors for each animal were used to determine plasma and CSF exposures over the first 24-h period (i.e., area under the curve from 0 to 24 h [AUC_0–24_]) using the “makeAUC” function within Pmetrics (Los Angeles, CA) (33, 36). The highest predicted concentration [*C*_max 0–24_] from the 12-min interval Bayesian estimates was determined to be each animal’s *C*_max 0–24_. The estimated cefepime PK exposures (AUC_0–24_ and *C*_max 0–24_) for plasma and CSF were used to calculate the percentage of penetration into the CSF for each animal (13–15). *T*_max_ and *t*_1/2_ were obtained from the “makeNCA” function in Pmetrics as applied to the first 8-h period of Bayesian predicted concentrations. *t*_1/2_ was calculated as ln(2)/*k* for the last 3 predicted concentrations. Summary statistics were calculated using GraphPad Prism version 7.02 (GraphPad Software, Inc., La Jolla, CA). PK exposure measure variability was calculated as the coefficient of variation percentage (CV%).

The estimated cefepime PK exposures (AUC_0–24_ and *C*_max 0–24_) for plasma and CSF were used to calculate the percentage of penetration into the CSF for each animal. This method was used similar to previous studies (13–15, 25). Ratios of the estimated AUC_CSF_/AUC_plasma_ and *C*_max CSF_/*C*_max plasma_ were used for percentage of CSF penetration. Only animals with CSF concentrations sampled were used for estimation of CSF penetration.
